# Dietary Influence on Urolithiasis Risk Mediated by Plasma Metabolites: A Mendelian Randomization and Experimental Study Linking Genes, Metabolites, and Clinical Outcomes

**DOI:** 10.1002/fsn3.70800

**Published:** 2025-08-19

**Authors:** Dawei Wang, Donghui Shi, Shuxin Si, Rao Xu, Fangxiu Luo, Zijian Zhou

**Affiliations:** ^1^ Department of Urology, Ruijin Hospital Shanghai Jiao Tong University School of Medicine Shanghai China; ^2^ Department of Urology Suzhou Wu Zhong People's Hospital Suzhou China; ^3^ Department of Urology, the First Affiliated Hospital of Fujian Medical University Fuzhou China; ^4^ Department of Rehabilitation Medicine The Affiliated Suqian First People's Hospital of Nanjing Medical University Suqian China; ^5^ Department of Pathology, Ruijin Hospital Shanghai Jiao Tong University School of Medicine Shanghai China; ^6^ Department of Urology, Huashan Hospital Fudan University Shanghai China

**Keywords:** food habit, gene‐metabolite interaction, mendelian randomization, targeted metabolomics, urolithiasis

## Abstract

Urolithiasis is a common and recurrent condition with significant health burdens; however, the causal relationships between dietary factors, plasma metabolites, and the urolithiasis risk remain poorly understood. To address this, we aimed to identify the causal associations between food habits and the risk of urolithiasis, and quantify the mediating role of plasma metabolites, employing two‐sample Mendelian randomization (MR) and experimental approaches. This MR analysis was based on summary statistics for calculus of kidney and ureter from published genome‐wide association studies (GWAS), including 10,556 cases and 400,681 controls of European ancestry. Furthermore, we used a two‐step MR to quantify the proportion of the effect of 1400 plasma metabolites‐mediated food habits on urolithiasis. Our MR analysis identified eight food intake factors and 15 food liking factors associated with urolithiasis. Metabolomic‐wide MR analysis identified 50 plasma metabolites associated with urolithiasis. Seven pairs of stone‐associated food factors and their metabolites were identified. Consistent with the MR results, widely targeted metabolomics analysis revealed that Mannose (fold change, FC = 0.54, *p* = 0.001) and Threonate (FC = 0.64, *p* = 0.0269) levels were significantly decreased in calcium oxalate (CaOx) kidney stone rat models compared with the control group. Integration of GWAS and eQTL data revealed 21 metabolite‐related genes using a summary‐data‐based MR (SMR) test. Analysis of GSE73680 revealed that LAMA2 (logFC = −1.31, *p* = 0.003) and CSNK1G3 (logFC = −0.76, *p* = 0.042) were downregulated in the CaOx group. Both genes (LAMA2: OR = 0.74, 95% CI = 0.56–0.98, *p* = 0.04; CSNK1G3: OR = 0.74, 95% CI = 0.58–0.94, *p* = 0.01) were associated with a reduced risk of CaOx stones. RNA‐Seq and RT‐qPCR assays validated that the expression of LAMA2 and CSNK1G3 was decreased in oxalate‐induced HK‐2 and NRK‐52E cells. Our study identified a causal relationship between food habits and the risk of urolithiasis with the effect mediated by plasma metabolites.

## Graphical abstract

This Mendelian randomization (MR) study identified causal links between 8 food intake factors, 15 food liking factors, and kidney stone risk, with 50 plasma metabolites mediating these associations. Experimental validation in rat models and renal cells revealed decreased Mannose/Threonate levels and downregulated LAMA2/CSNK1G3 genes in stone formation. These findings highlight diet‐driven kidney stone risk, partially mediated by metabolites and genetic pathways.

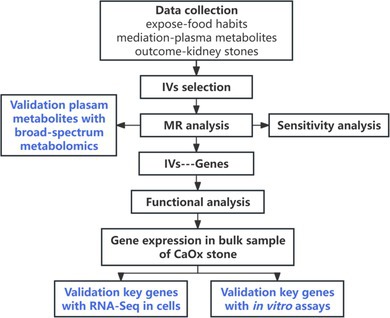

**Abbreviations**
CI: Confidence interval
GWAS: Genome‐wide association study
IV: Instrumental variable
IVW: Inverse‐variance weighted
LD: Linkage disequilibrium
MR: Mendelian randomization
OR: Odds ratio
MR‐PRESSO: Mendelian randomization pleiotropy residual sum and outlier
SD: Standard deviation
SNP: Single nucleotide polymorphism

## 1 Introduction

Urolithiasis is a highly prevalent disease with an increasing global incidence, posing a significant health burden due to associated morbidity, healthcare costs, and reduced quality of life (Sorokin et al. 2017). This condition is characterized by the formation of solid crystalline deposits within the renal system, which can cause severe pain, urinary obstruction, and kidney damage in extreme cases (Jin et al. 2025). Among the various subtypes of kidney stones, calcium oxalate (CaOx) stones are the most common, accounting for approximately 80% of cases worldwide (Kachkoul et al. 2023). Despite advances in understanding the pathophysiology of urolithiasis, the precise mechanisms linking dietary factors, systemic metabolic changes, and genetic predisposition to stone formation remain poorly understood (X. Wang and Wang 2024). Currently, preventative treatments such as thiazide diuretics to reduce hypercalciuria and alkaline citrate to manage hyperoxaluria are used to minimize stone recurrence (Lyall, Wood, & Pais, 2023). However, evidence supporting their effectiveness is not entirely conclusive, which highlights the need for a deeper understanding of the pathological processes underlying stone formation.

The composition of the diet is recognized as a significant factor in stone formation (Siener et al. 2021). Beverages like tap water, mineral water, fruit juices, soft drinks, tea, and coffee have been debated for their effects on urolithiasis (Barghouthy et al. 2021a; Ferraro et al. 2013; Peerapen and Thongboonkerd 2018). Additionally, dietary components, including proteins, carbohydrates, oxalate, calcium, and sodium chloride, influence urinary risk profiles, thereby contributing to the risk of urolithiasis (Liu et al. 2020; Meschi et al. 2012). Current dietary recommendations focus on reducing lithogenic risk factors to decrease urine supersaturation, particularly for CaOx, calcium phosphate (CaP), and uric acid stones by advocating fluid intake (Robertson 2016; Yitgin et al. 2023). Scientific evidence consistently highlights the adverse effects of high meat/animal protein consumption and low calcium diets, while diets rich in fruits, vegetables, and balanced low‐fat dairy products pose the lowest risk for urolithiasis (Ferraro et al. 2020). Despite these findings, the relationship between dietary habits and urolithiasis risk has not been fully elucidated owing to the complexity of diet, dietary assessment limitations, heterogeneity of urolithiasis, interindividual variability, and observational and cross‐sectional studies.

Plasma metabolites include lipids, amino acids, sugars, and a wide range of other biochemical compounds that are small molecules in the blood, resulting from metabolic processes within the body (Bar et al. 2020). The composition of plasma metabolites is significantly influenced by dietary intake, thereby bridging the gap among nutrition, metabolism, and health outcomes (L. Chen et al. 2022; Pietzner et al. 2021). The interplay between diet, plasma metabolites, and urolithiasis risk is complex, as dietary intake affects the concentration and composition of plasma metabolites, which in turn modulates urolithiasis risk (Fakhoury et al. 2019; Marsh et al. 2019). Therefore, diet‐related metabolites linked to kidney stone risk may serve as effective surrogate markers to guide dietary interventions.

Unlike previous studies that focused on observational associations or single Mendelian randomization (MR), our research aimed to establish causality between food intake, food liking, and the risk of urolithiasis by a multi‐omics approach, combining MR, metabolomics, RNA‐sequencing (RNA‐Seq), bioinformatics, and experimental assays. Investigating the potential mediating role of plasma metabolites in the diet‐stone risk nexus is another critical question that our study aimed to address, offering mechanistic insights into how dietary factors influence stone formation. Furthermore, we utilized broad‐spectrum metabolomics and RNA‐Seq with bioinformatics to identify the key metabolites and genetic pathways associated with CaOx kidney stones. To ensure biological relevance, our study integrates in vivo and in vitro validation of key metabolites and genes, providing a strong foundation for targeted dietary interventions and advancing the understanding of CaOx kidney stones.

## 2 Materials and Methods

### 2.1 Study Design

**FIGURE 1:**
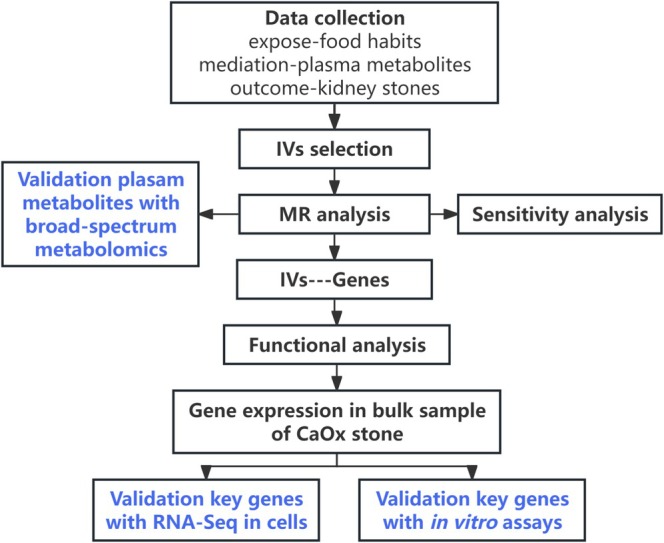
The schematic study design.

Figure 1 illustrated the schematic of the study design. Firstly, we performed MR analyses to explore the calculus of kidney and ureter‐associated food intake and food liking factors. Likewise, we conducted a metabolomic‐wide MR analyses (which involves using genetic variants to estimate the causal effects of exposure on clinical outcomes) to identify the calculus of kidney and ureter‐associated plasma metabolites. In the metabolomic‐wide MR on the calculus of kidney and ureter, we identified appropriate genetic variants as instrumental variables (IVs) to simulate plasma metabolite levels, utilizing data from the genome‐wide association study (GWAS) Catalog (https://www.ebi.ac.uk/gwas/). Subsequently, we employed the MR approach to estimate the associations between plasma metabolites and the risk of calculus of kidney and ureter. Next, using a two‐stage network MR analysis, we estimated the mediation effects of plasma metabolites on the associations between food habits and the risk of calculus of kidney and ureter. Following the identification of relevant metabolites, we performed the additional downstream analyses: (1) Mapping single nucleotide polymorphisms (SNPs) to their closest gene, (2) Summary‐data‐based MR (SMR) analysis, (3) Functional enrichment and bulk RNA data analysis of GSE73680, and (4) RNA‐Seq and in vitro assays. This study was conducted in accordance with the Strengthening the Reporting of Observational Studies in Epidemiology using Mendelian Randomization (STROBE‐MR) guidelines (Skrivankova et al. 2021).

### 2.2 Data Sources

Food intake and food liking: The genetic data for the 38 food intake factors and 187 food liking factors used in this study were obtained from the latest GWAS summary data conducted by May‐Wilson et al. (2022) and Pirastu et al. (2022). In short, they performed a systematic GWAS analysis of food intake in 445,779 UK Biobank participants (Table S1) and food liking in 161,625 UK Biobank participants (Table S2). Calculus of kidney and ureter: Data on calculus of kidney and ureter were derived from a large prospective cohort study in Finland, FinnGen (Round 10), including 10,556 cases and 400,681 controls (Kurki et al. 2023). Plasma metabolites: In the recent study, a GWAS was conducted on 1400 plasma metabolites implicated in human diseases (Chen et al. 2023). The data can be accessed from the GWAS Catalog database, which includes catalog numbers for the 1400 plasma metabolites (GCST90199621‐GCS90201020, Table S3). The selection of IVs for two‐sample MR followed the following criteria: (1) A significance threshold for each blood cell perturbation response data within the locus range was set at *p* < 5.0 × 10^−6^ (Kwok and Schooling 2021; Mu et al. 2024); (2) The 1000 Genomes European reference panel was used to calculate linkage disequilibrium (LD) between SNP with the threshold of *r* ^2^ < 0.001 and kb = 10,000, prioritizing SNPs with lower *P*‐values; (3) Palindromic SNPs were eliminated; and (4) SNPs with an *F*‐statistic < 10 were excluded to avoid weak IV bias.

### 2.3 MR Statistical Analysis

For the MR and reverse MR analyses, we employed the inverse‐variance weighted (IVW) method with multiplicative random effects as the primary analytical approach because of its high statistical power. This was complemented by three sensitivity analyses, prioritizing SNPs selected at the stringent threshold of *p* < 5 × 10^−8^, including the weighted median, MR‐Egger, and Mendelian randomization pleiotropy residual sum and outlier (MR‐PRESSO) to assess the robustness of the results and detect potential horizontal pleiotropy (Yan et al. 2024). To evaluate the heterogeneity among the SNPs used as IVs, we used the Cochrane's Q test. The Stepwise test method was utilized to estimate the effects associated with the mediation.

### 2.4 Mapping SNPs to Genes

We utilized the “vautils” R package to map each queried variant to its closest gene, which could be an overlapping gene or a downstream or upstream gene. To ensure the accuracy of genomic locations, IV SNPs for plasma metabolites potentially causally associated with the calculus of kidney and ureter were used with the hg38 version of the genomic data.

### 2.5 Summary‐Data‐Based MR (SMR) Analysis

SMR analysis was utilized as an additional method to confirm the causal relationships between genes related to plasma metabolites and the risk of calculus of kidney and ureter (Wu et al. 2018). The heterogeneity in dependent instruments (HEIDI) test, which employs multiple SNPs within a specific region, was used to differentiate genes influencing the risk of calculus of kidney and ureter due to a common genetic variant rather than through genetic linkage. The SMR and HEIDI tests were performed using SMR software (SMR v1.3.1). The overall level statistics for eQTLs were derived from the eQTLgen Consortium (Võsa et al. 2021), which analyzed gene expression at the transcript level in peripheral blood from 31,684 individuals, predominantly of European ancestry.

### 2.6 CaOx Kidney Rat Models and Histologic Analysis

Sprague–Dawley (SD) rats, aged 6 weeks, were used to develop CaOx nephrolithiasis models. 12 male rats were divided into a control group (named Con), which received sterile tap water, and a model group (named CaOx), which received 1% ethylene glycol (EG) in their drinking water for 4 weeks. Following this period, samples of blood plasma and renal tissues were collected. Renal function was assessed using an automatic biochemical analyzer to measure creatinine (CREA) and blood urea nitrogen (BUN). Histological analyses, including hematoxylin & eosin (HE), Von Kossa (VK), and alizarin red (AR) staining, were conducted to confirm the model establishment and evaluate renal injury.

### 2.7 Widely Targeted Metabolomics

Plasma metabolites were extracted by the following protocol: 50 μL aliquot of the plasma was mixed with a methanol/acetonitrile/water mixture (2:2:1, v/v) under sonication in ice baths for 1 h, followed by incubation at −20°C for 1 h, and centrifugation at 16,000 g, 4°C for 20 min; then the supernatant was used for analysis. Quality control (QC) samples, prepared by pooling aliquots from all experimental samples, were used for data normalization. The extracts were dissolved in 50% acetonitrile, filtered, and stored at −80°C until analysis. Ultra‐high‐performance liquid chromatography coupled with mass spectrometry (UHPLC–MS) was performed using a Shimadzu Nexera X2 system and a 5500 QTRAP mass spectrometer (Sarfaraz et al. 2016). This widely targeted metabolomics study was conducted by Bioprofile (Shanghai, China). The separation was achieved on an Acquity UPLC HSS T3 column with a gradient of 0.1% formic acid (solvent A) and acetonitrile (solvent B). Metabolites were detected in both the positive and negative ionization modes. Data were preprocessed with MultiQuant 3.0.2 to obtain peak areas, and discriminating metabolites were identified using variable influence on projection (VIP) values from Orthogonal Partial Least Squares Discriminant Analysis (OPLS‐DA) models and Student's t‐test with *P*‐values corrected for multiple testing via the Benjamini‐Hochberg method with false discovery rate (FDR) < 0.05. Metabolites with VIP > 1 and *p* < 0.05 were considered statistically significant and further analyzed using cluster analysis with the R package.

### 2.8 Bulk RNA Data Analysis of GSE73680

We downloaded the data of GSE73680 from the Gene Expression Omnibus (GEO) database (https://www.ncbi.nlm.nih.gov/geo/) (Taguchi et al. 2017). 24 CaOx Randall's Plaque papillary tissues from CaOx stone formers and six normal papillary tissues from control patients were used to generate the GSE73680 dataset. The “limma” R package was used to identify differentially expressed genes (DEGs) between six normal papillary tissues (named Control) and 24 CaOx Randall's Plaque papillary tissues (named Randall's Plaque). To address the high false positive rate inherent in testing 1000 of genes simultaneously, *P*‐values were adjusted for multiple testing using the Benjamini‐Hochberg method to control the FDR. Genes with *P*.adj < 0.05 were considered significantly differentially expressed.

### 2.9 Pathway Enrichment Analysis of LAMA2/CSNK1G3 and Mannose/Threonate

The molecular structures of Mannose and Threonate were retrieved from the PubChem database (https://pubchem.ncbi.nlm.nih.gov/). The corresponding SDF files of the 3D structures were then uploaded to the PharmMapper database (http://lilab.ecust.edu.cn/pharmmapper/) to predict the potential human protein targets associated with Mannose and Threonate. Subsequently, the predicted protein targets were converted into their corresponding gene symbols using the UniProt database (https://www.uniprot.org/). A Venn diagram was used to identify common target genes of Mannose and Threonate. To identify the pathways associated with LAMA2/CSNK1G3, data were sourced from the Comparative Toxicogenomics Database (CTD, https://ctdbase.org/). Additionally, a protein–protein interaction (PPI) network was constructed using the STITCH database (http://stitch.embl.de). To further explore the biological roles and signaling pathways of these target genes, Gene Ontology (GO) and Kyoto Encyclopedia of Genes and Genomes (KEGG) pathway enrichment analyses were performed using “clusterProfiler” and “enrichplot” R packages with adjusted *p* < 0.05 after using the Benjamini‐Hochberg method to control FDR.

### 2.10 Cell Culture, Cell Proliferation, and Viability Assays

Human kidney 2 (HK‐2) cells and normal rat kidney (NRK‐52E) cells were procured from the Chinese Academy of Sciences (Shanghai, China). Cells were exposed to varying concentrations of oxalate (0, 0.5, 1, 1.5, 2, and 3 mM) for 24 h (Ke et al. 2024; Zhou et al. 2023). After treatment with graded concentrations of oxalate, cell proliferation was quantified by adding 10 μL of Cell Counting Kit‐8 (CCK‐8) solution (Servicebio, Wuhan, China) to each well, and the absorbance was measured at 450 nm. Lactate dehydrogenase (LDH) activity, an indicator of cellular damage, was determined using an LDH Assay Kit (Beyotime, Shanghai, China), and the absorbance was measured at 490 nm, according to the manufacturer's protocol.

### 2.11 Reactive Oxygen Species (ROS) Staining

The detection of ROS production was detected by 2,7‐dichlorodihydrofluorescein diacetate (DCFH‐DA) staining. For the DCFH‐DA staining protocol, cells subjected to treatment were incubated in serum‐free media containing 10 μM DCFH‐DA at 37°C for 30 min. After incubation, the cells were washed twice with serum‐free media; then ROS generation was visualized using a fluorescence microscope.

### 2.12 Flow Cytometry Apoptosis Assay

Apoptosis in HK‐2 and NRK‐52E cells was assessed via flow cytometry using an Annexin V‐Fluorescein Isothiocyanate (FITC) Apoptosis Detection kit (Servicebio, Wuhan, China) following the provided protocol. Flow cytometry (Becton Dickinson, USA) was used to determine the apoptotic cell rate.

### 2.13 RNA‐Sequencing and DEGs Analysis

Total RNA was extracted from HK‐2 and NRK‐52E cells using TRIzol reagent. Subsequent ribosomal RNA (rRNA) depletion was performed using the RiboGone rRNA Removal Kit. RNA quality and concentration were determined by Nanodrop spectrophotometry and SDS‐PAGE electrophoresis. The prepared library was then loaded onto a flow cell for cluster generation, with surface‐bound oligonucleotides complementary to the adapter sequences capturing the cDNA fragments. After cluster generation, the libraries were sequenced on an Illumina HiSeq 4000 platform. *P*‐values were adjusted for multiple testing using the Benjamini‐Hochberg method to control for FDR. The DEGs were identified according to predetermined threshold criteria: *P*.adj < 0.05 and |fold change| > 2.

### 2.14 RT‐qPCR Assays

Total RNA was extracted with the RNA Sample Total Kit (NCM Biotech, Suzhou, China), following the manufacturer's instructions, and cDNA synthesis was performed subsequently. RT‐qPCR was carried out in triplicate using the SYBR Green PCR Kit (Vazyme, Nanjing, China), with primers of LAMA2 and CSNK1G3 listed in Table S4.

### 2.15 Statistical Analysis

Statistical analyses were performed using R software (version 4.3.0) and GraphPad Prism (version 9.5). For the MR analysis of gene‐calculus of kidney and ureter associations, the odds ratio (OR) and corresponding confidence interval (CI) of the association were estimated by the Wald ratio test and the delta method, respectively. The significance threshold was set at a *P*‐value < 0.05.

## 3 Results

### 3.1 Eight Food Intake Factors and 15 Food Liking Factors Were Associated With Calculus of Kidney and Ureter

**FIGURE 2:**
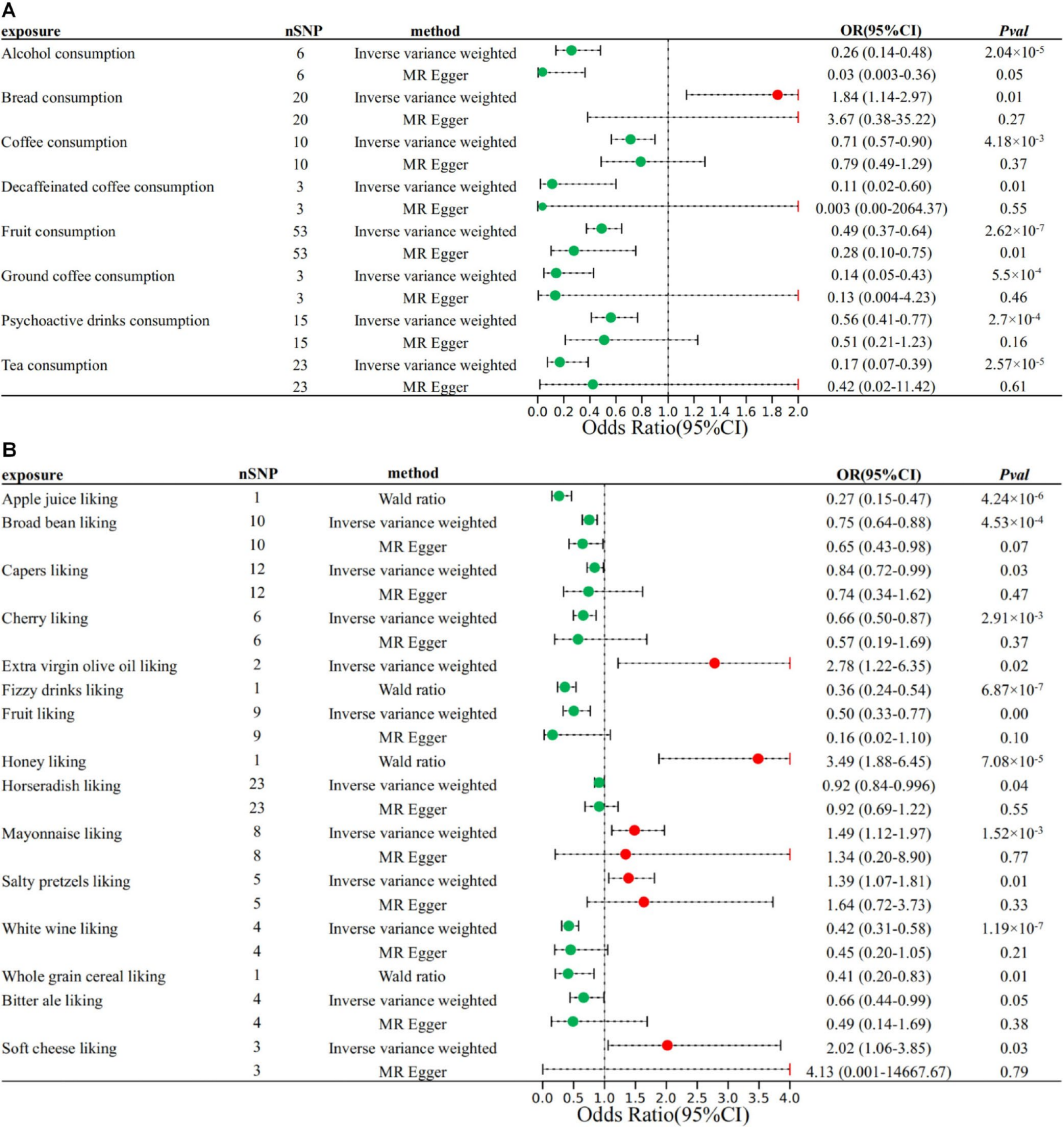
Food habits were associated with calculus of kidney and ureter. (A, B) Forest plot of the MR analysis of food intake (A) and food liking (B) factors as the exposure and calculus of kidney and ureter as the outcome.

Genetically proxied eight out of 38 food intake factors were associated with calculus of kidney and ureter (Figure 2A). Genetic predisposition to bread consumption was associated with an increased risk of calculus of kidney and ureter. Genetic predisposition to coffee consumption, psychoactive drinks consumption, fruit consumption, alcohol consumption, tea consumption, and other factors were associated with a decreased risk of calculus of kidney and ureter. These associations were consistent in the sensitivity analyses, which were evaluated by pleiotropy and heterogeneity. No significant heterogeneity was observed (*P*‐value > 0.05, Table S5), and no indication of horizontal pleiotropy was observed with the MR‐Egger intercept values (*P*‐value > 0.05, Table S6).

After removing metabolites without SNPs in the outcome data or with weak instruments (*F‐*statistic < 10), the MR analysis included a total of 187 food liking factors. Genetically proxied 15 out of 187 food intake factors were associated with calculus of kidney and ureter (Figure 2B). Genetic predisposition to salty pretzels liking, mayonnaise liking, soft cheese liking, extra virgin olive oil liking, and honey liking was associated with an increased risk of calculus of kidney and ureter. Genetic predisposition to apple juice liking, fizzy drinks liking, whole grain cereal liking, white wine liking, and other factors was associated with a decreased risk of calculus of kidney and ureter. No significant heterogeneity was observed (*P*‐value > 0.05, Table S7), and no indication of horizontal pleiotropy was observed with the MR‐Egger intercept values (*P*‐value > 0.05, Table S8).

### 3.2 Metabonomic‐Wide MR Analysis Identified Hub Plasma Metabolites Associated With Calculus of Kidney and Ureter

**FIGURE 3:**
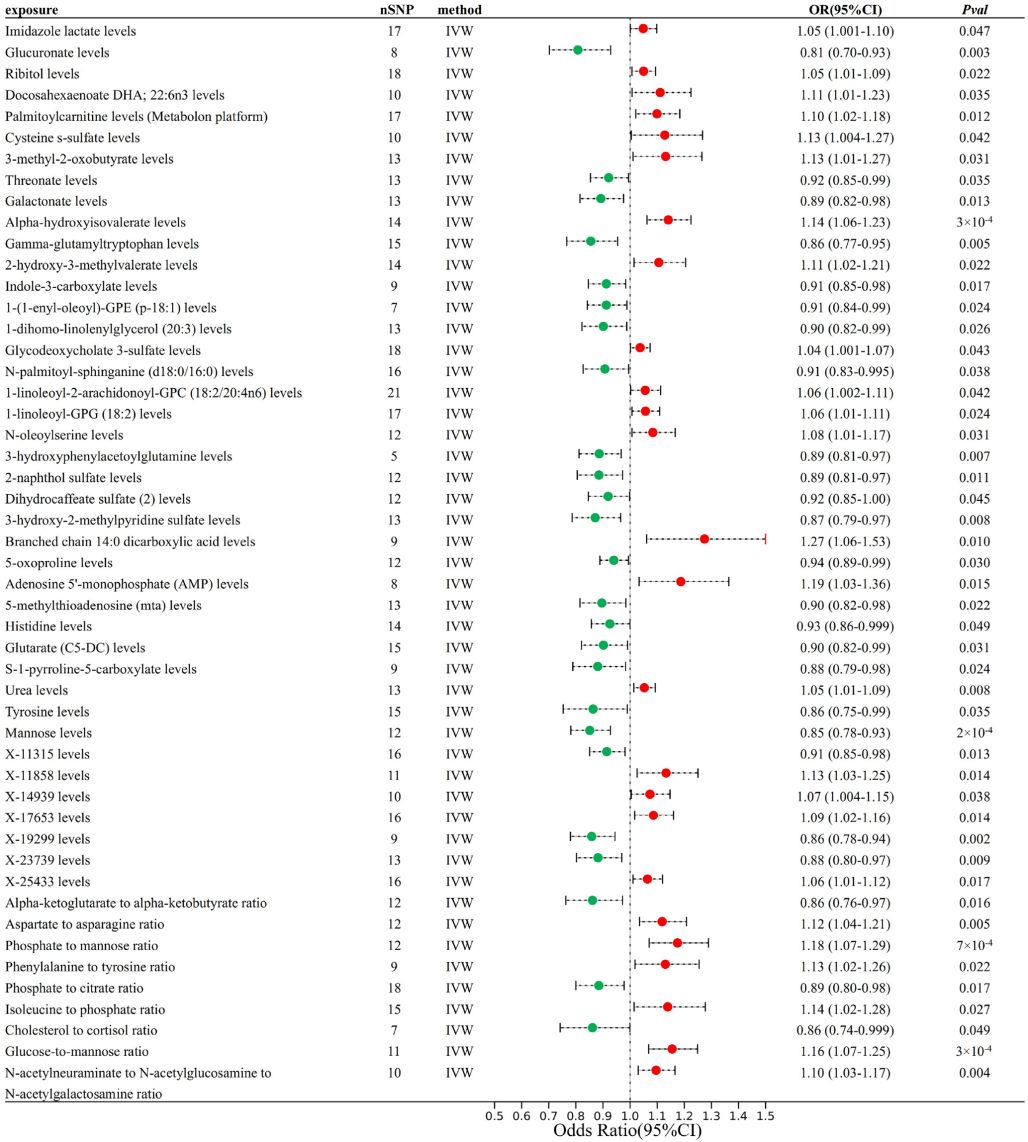
Forest plot of the MR analysis of plasma metabolites as the exposure and calculus of kidney and ureter as the outcome.

After removing metabolites without SNPs in the outcome data or with weak instruments (*F*‐statistic < 10), the metabonomic‐wide MR analysis included a total of 1400 plasma metabolites (Table S9). With the significance threshold set to 0.05, we selected the values of metabolites consistent with the IVW method as plasma metabolites with a significant causal relationship with calculus of kidney and ureter (Table S10). The MR‐PRESSO analysis indicated outliers for the IV SNPs for Tyrosine levels and Phosphate to citrate ratio, but the Egger intercept analysis found no evidence of horizontal pleiotropy. Among them, tyrosine levels and phosphate to citrate ratio were excluded because of the presence of heterogeneity and pleiotropy. Finally, 50 plasma metabolites with a robust causal relationship to kidney calculus were obtained (Figure 3).

### 3.3 Associations Between Calculus of Kidney and Ureter‐Associated Food Factors and Calculus of Kidney and Ureter‐Associated Metabolites

**FIGURE 4:**
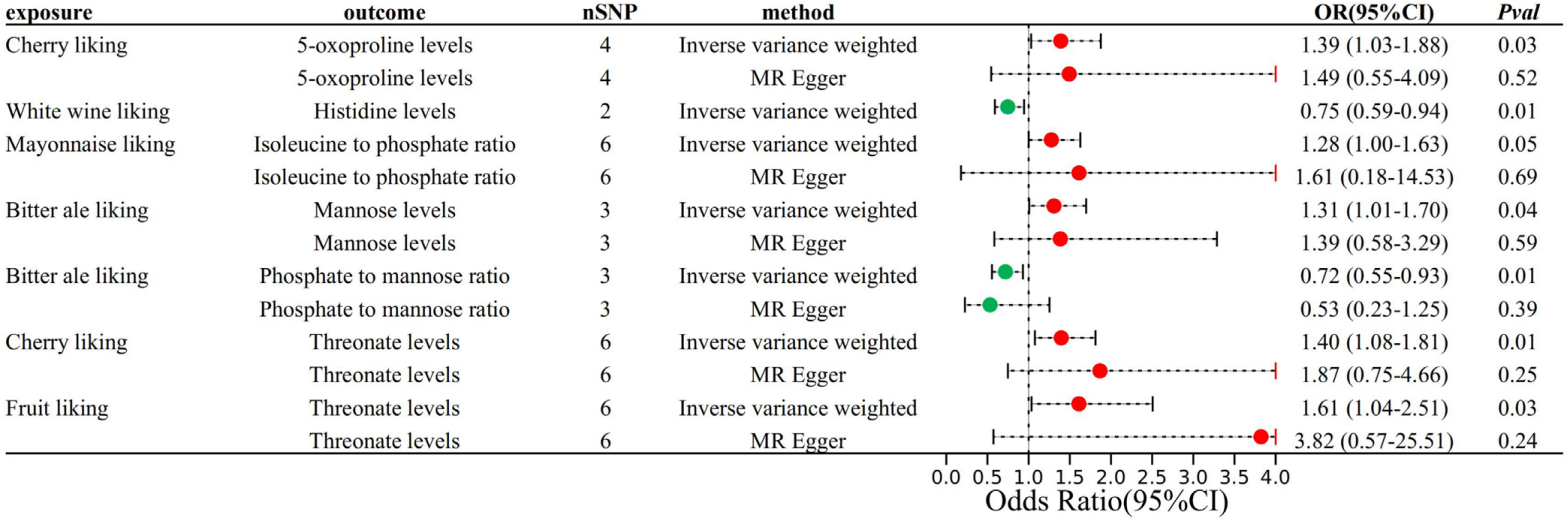
Forest plot of the MR analysis of eight food intake factors, 15 food liking factors, and 50 plasma metabolites.

In the MR analyses of the associations between calculus of kidney and ureter‐associated food factors and calculus of kidney and ureter‐associated metabolites, we set the significance level at the nominal level to reveal as many potential mediation signals as possible. In total, seven pairs of associations between calculus of kidney and ureter‐associated food factors and metabolites were identified (Table S11). Among calculus of kidney and ureter‐associated food factors, genetically predicted Cherry liking indicators were associated with 5‐oxoproline levels (OR = 1.39, 95% CI: 1.03–1.88, *p* = 0.03) and Threonate levels (OR = 1.40, 95% CI: 1.08–1.81, *p* = 0.01); White wine liking indicators were associated with Histidine levels (OR = 0.75, 95% CI: 0.59–0.94, *p* = 0.01); Bitter ale liking indicators were associated with Mannose levels (OR = 1.31, 95% CI: 1.01–1.70, *p* = 0.04) and Phosphate to mannose ratio (OR = 0.72, 95% CI: 0.55–0.93, *p* = 0.01); Fruit liking indicators were associated with Threonate levels (OR = 1.61, 95% CI: 1.04–2.51, *p* = 0.03, Figure 4). To avoid potential pleiotropic effects, we conducted a multivariate MR (MVMR) analysis (Table S12). The MVMR analysis revealed that Bitter ale liking was significantly associated with a reduced risk of kidney stones using the IVW method (Beta = −0.4119, *p* = 0.00096, OR = 0.6624, 95% CI: 0.5187–0.8458), with consistent findings from the Egger (*p* = 0.00089) and median methods. Similarly, Mayonnaise liking showed a significant positive association with kidney stone risk using the IVW method (Beta = 0.3181, *p* = 0.0135, OR = 1.3745, 95% CI: 1.0680–1.7690). These results highlight the potential causal relationships between dietary preferences and kidney stone risk.

### 3.4 Mediation of Plasma Metabolites in the Associations Between Food Factors and Calculus of Kidney and Ureter

**FIGURE 5:**
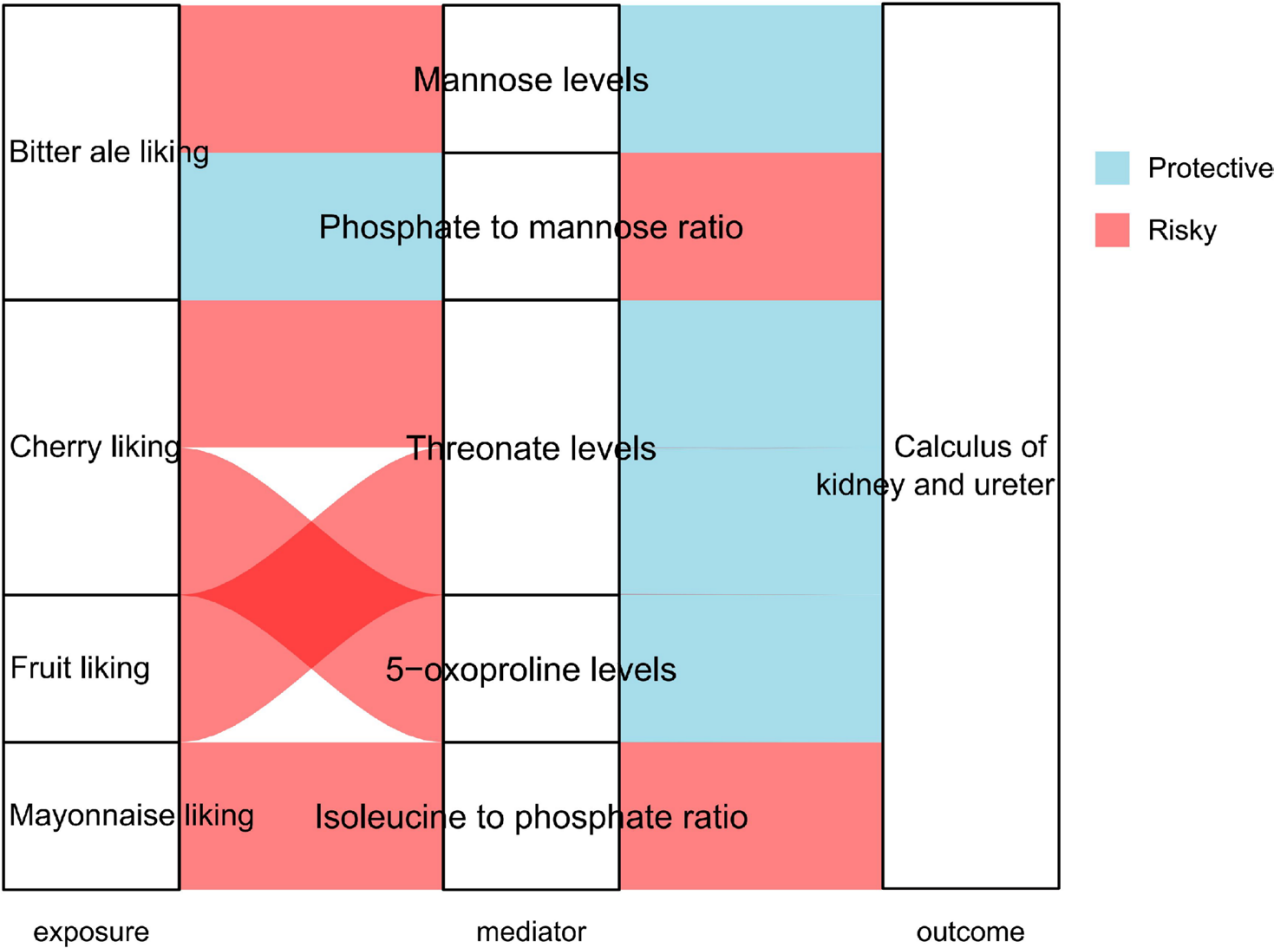
Sankey plot of the mediation associations in food factors, plasma metabolites, and calculus of kidney and ureter.

We estimated the mediation of six food factor‐metabolite‐calculus combinations where the direction of the total effect (β of the food factor‐calculus association) was in line with the direction of the effect through the mediator (β of the food factor‐metabolites association × β of the metabolites‐calculus association) (Table S13). Two of the six combinations were related to the bitter ale liking indicator, and two metabolites (Mannose levels [mediation effects: 10.50%], and Phosphate to mannose ratio [mediation effects: 13%]) showed mediation effects on the associations (Miao et al. 2025) with calculus of kidney and ureter risk (Figure 5). Genetically predicted levels of threonate mediated the association between fruit liking and calculus of kidney and ureter [mediation effects: 5.7%]. Likewise, genetically predicted levels of threonate [mediation effects: 6.5%] and 5‐oxoproline levels [mediation effects: 4.85%] mediated the association between cherry liking traits and calculus of kidney and ureter. Genetically predicted levels of isoleucine to phosphate ratio [mediation effects: 8%] mediated the association between mayonnaise liking traits and calculus of kidney and ureter. No evidence of reverse causality between food factors and kidney stones was found (Table S14).

### 3.5 Validation of Plasma Metabolites in EG‐Induced CaOx Kidney Stone Rat Models

**FIGURE 6:**
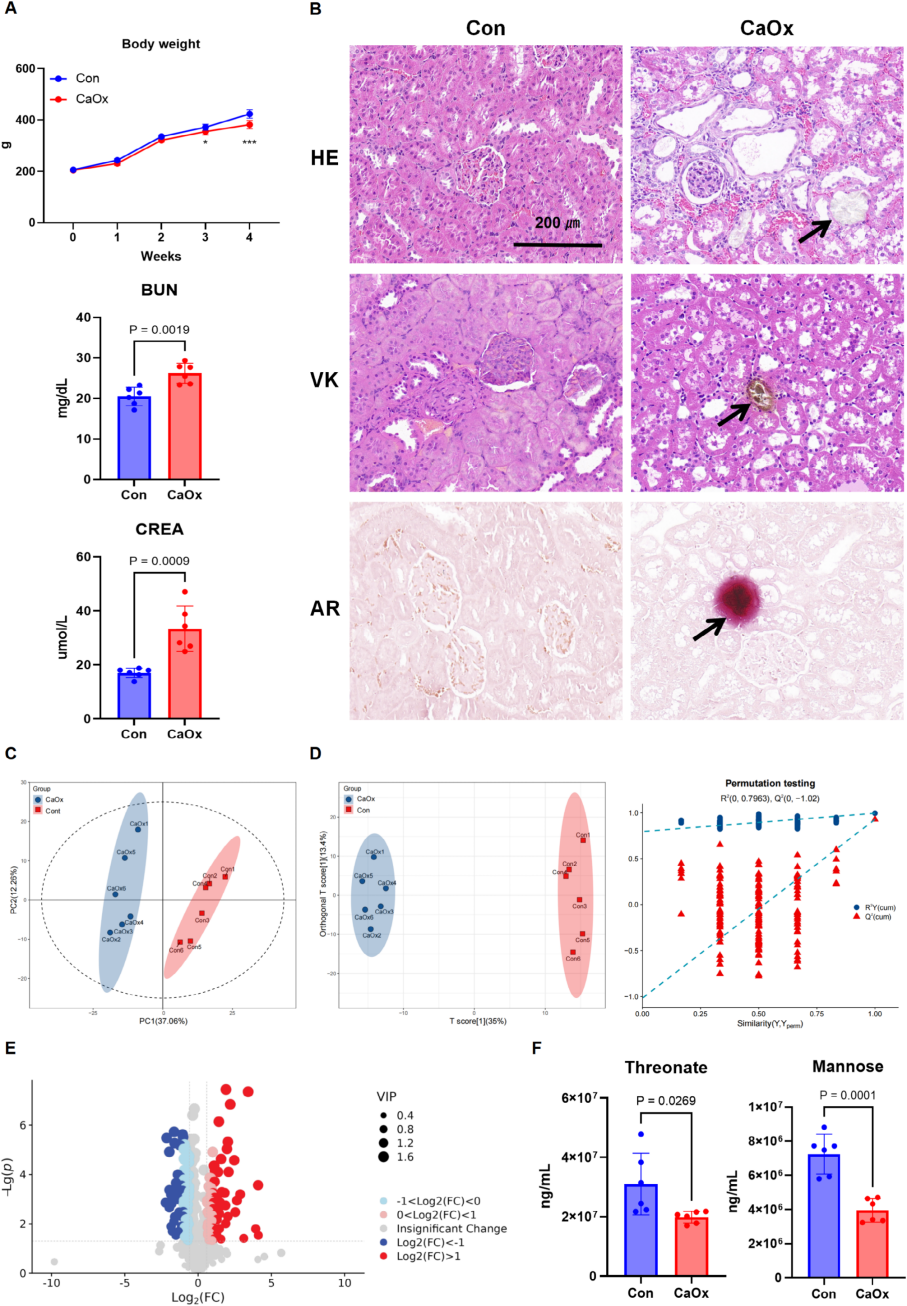
Validation key plasma metabolites in CaOx kidney stone rat models. (A) The body weight, CREA, and BUN levels of rats. (B) Pathological sections including HE, VK, and AR staining showed the degree of CaOx crystals and kidney injury (magnification×20; scale bar, 200 μm). (C, D) The PCA plot (C), OPLS‐DA scores and permutation tests (D) based on widely targeted metabolomics of the plasma metabolites in rats. (E) The volcano plot of differential metabolites between the groups. (F) The levels of Threonate and Mannose were decreased in CaOx kidney stones.

Next, we validated the above plasma metabolites in EG‐induced CaOx kidney stone rat models. The CaOx group exhibited decreased body weight, along with significantly elevated levels of creatinine (CREA) and blood urea nitrogen (BUN) compared to the Con group (Figure 6A). Histological analyses revealed crystal deposition, tubular dilation, atrophy, interstitial inflammation, and kidney injury in the CaOx group, confirming the successful establishment of the model (Figure 6B). Metabolomic profiling using PCA (Figure 6C) and OPLS‐DA (Figure 6D) models demonstrated clear distinctions between the control and CaOx groups, with 287 significantly different metabolites identified (Figure 6E). Consistent with the MR analysis, the Mannose and Threonate levels were significantly decreased in the CaOx group, indicating a low risk of kidney stones (Figure 6F). These findings underscore the reliability of the experimental model and highlight the significant alterations in the metabolomic profiles associated with CaOx‐induced renal injury.

### 3.6 Genes and Functions

**FIGURE 7:**
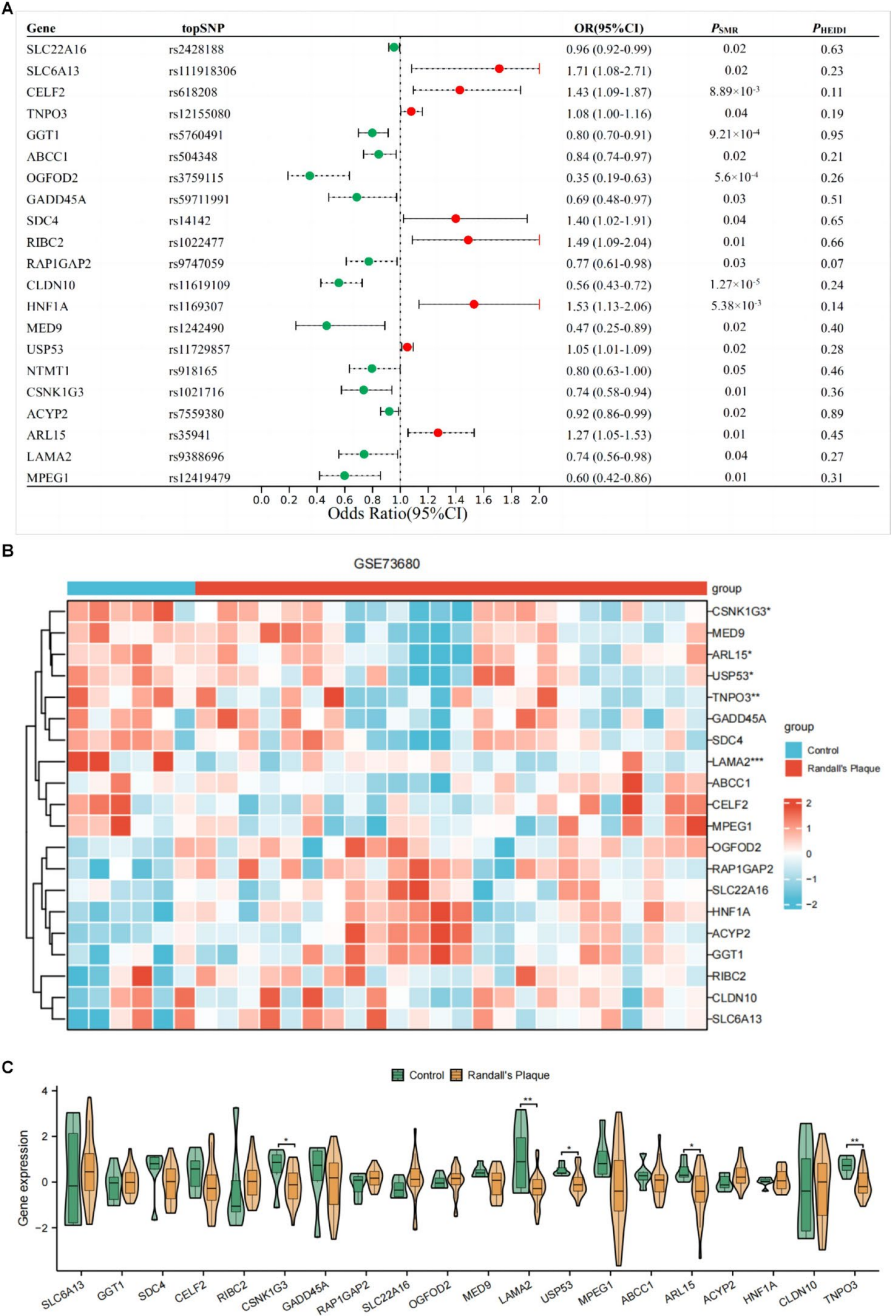
IVs neighboring genes combined bulk RNA analysis of GSE73680. (A) SMR analysis showing the IVs neighboring genes with causal association with kidney stones. (B, C) Heatmap (B) and boxplots (C) showing the differential expression of CaOx kidney stone‐related genes in patients with CaOx kidney stones versus normal tissues in GSE73680.

Based on the IVs of the 50 hub metabolites, 531 neighboring genes were identified (Table S15). To explore the causal relationship between these metabolite‐related genes and calculus of kidney and ureter, we performed SMR analysis using eQTL data from the eQTLgen Consortium, and 21 genes related to calculus of kidney and ureter were found, including CLDN10, OGFOD2, GGT1, MPEG1, HNF1A, CELF2, ARL15, RIBC2, CSNK1G3, LAMA2, and others (Figure 7A).

### 3.7 Bulk RNA Analysis of GSE73680

Within the GSE73680 dataset, we performed differential gene analysis of the 21 genes from the SMR analysis between control individuals and CaOx stone patients (Table S16). We identified five significant genes that downregulated LAMA2, TNPO3, CSNK1G3, ARL15, and USP53 in CaOx patients compared to those in control individuals (Figure 7B,C, Table S17). Among them, the downregulation of LAMA2 and CSNK1G3 in CaOx patients from the GSE73680 dataset was consistent with the results of the SMR analysis. Therefore, our analysis indicated that LAMA2 and CSNK1G3 are key genes in CaOx kidney stones.

### 3.8 Mechanistic Connection of LAMA2 and CSNK1G3 to Mannose/Threonate

The 2D molecular structures of Mannose/Threonate were obtained from the PubChem database (Figure S1A). A total of 221 common target genes of Mannose and Threonate were identified using the PharmMapper database (Figure S1B). Pathway enrichment analysis was performed to elucidate the biological roles and pathways of the target genes for Mannose and Threonate (Figure S1C). Next, a protein–protein interaction (PPI) network for LAMA2 and CSNK1G3 was generated using the STITCH database (Figure S1D). GO analysis revealed involvement in the regulation of cell–substrate adhesion, regulation of cell–matrix adhesion, and cell–substrate adhesion in the biological process (BP) category (Figure S1E), which was similar to the pathways of the target genes for Mannose and Threonate. KEGG analysis indicated that these genes were primarily involved in ECM–receptor interaction, Focal adhesion, and PI3K‐Akt signaling pathway (Figure S1E). Similarly, the results from the Comparative Toxicogenomics Database (CTD) indicated that the LAMA2 gene is primarily involved in the following pathways: ECM–receptor interaction, focal adhesion, PI3K‐Akt signaling pathway, and extracellular matrix organization (Figure S1F). These pathways are similar to the pathways enriched by the target genes for Mannose and Threonate.

### 3.9 Validation of LAMA2 and CSNK1G3 Expression in Oxalate‐Induced Cells With RNA‐Seq and in Vitro Assays

**FIGURE 8:**
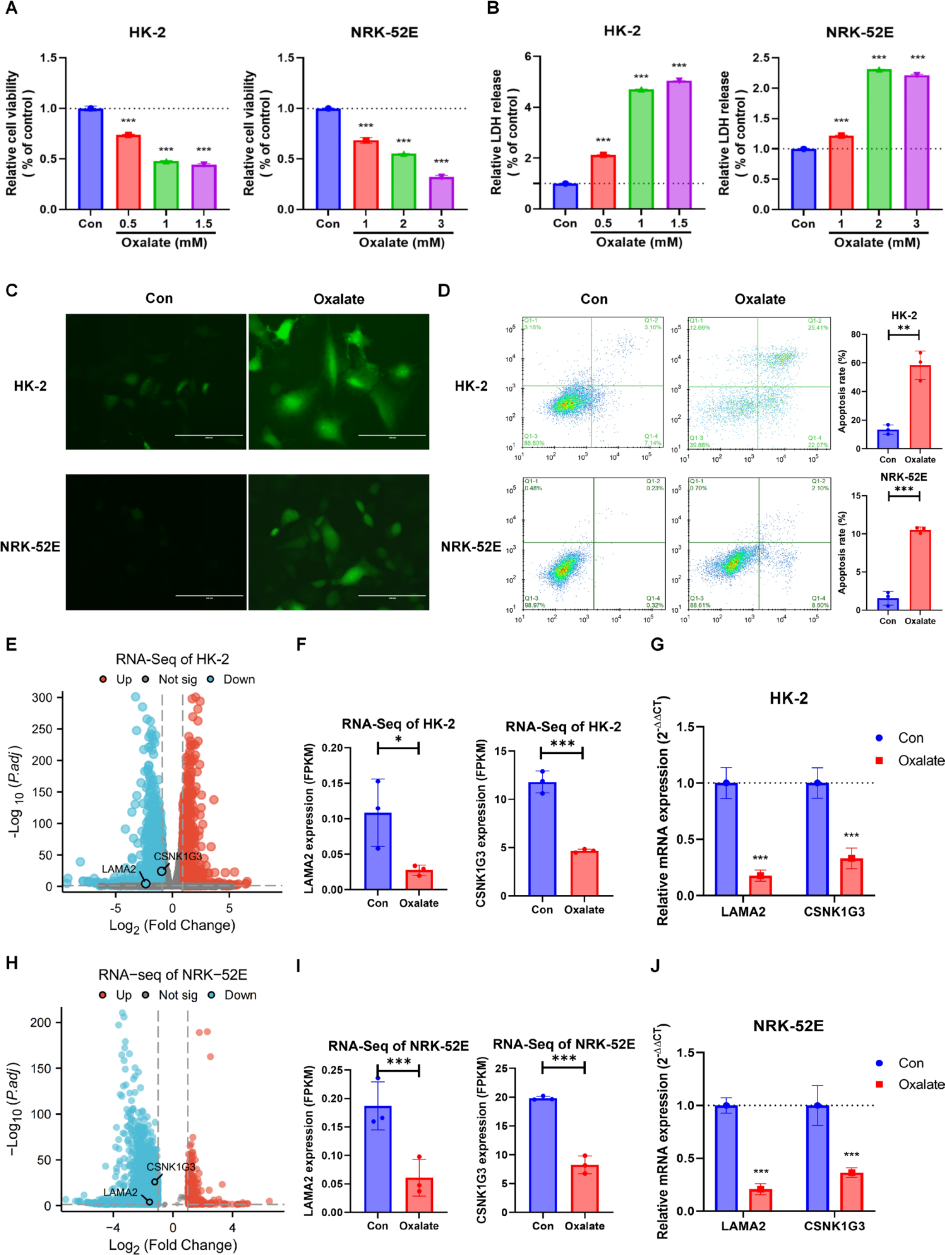
Validation LAMA2 and CSNK1G3 expression in oxalate‐induced HK‐2 and NRK‐52E cells. (A, B) Cell viability (A) and LDH release (B) after treatment with oxalate. (C) ROS staining was used to assess the effect of oxalate on cytoplasmic ROS generation in cells (scale bar, 100 μm). (D) Apoptotic rate of cells was determined by flow cytometry. (E, F) The volcano plot (E) and the bar graph (F) showing that LAMA2 and CSNK1G3 were downregulation in oxalate‐induced HK‐2 cells using RNA‐Seq. (G) RT‐qPCR validation of LAMA2 and CSNK1G3 expression in HK‐2 cells. (H, I) The volcano plot (H) and the bar graph (I) showing that LAMA2 and CSNK1G3 were downregulation in oxalate‐induced NRK‐52E cells using RNA‐Seq. (J) RT‐qPCR validation of LAMA2 and CSNK1G3 expression in NRK‐52E cells (Mean ± SD, *n* = 3, *p < 0.05, ***p < 0.001).

As determined by the CCK‐8 assay, it was revealed that oxalate significantly reduced the proliferation of HK‐2 and NRK‐52E cells in a concentration‐dependent manner (Figure 8A). In parallel, treatment with oxalate markedly elevated LDH release from HK‐2 and NRK‐52E cells, indicating increased cell damage in a concentration‐dependent manner (Figure 8B). Given the substantial cellular damage observed at different concentrations of oxalate, we chose a 1 mM oxalate level for subsequent treatments of HK‐2 cells and a 2 mM oxalate level for subsequent treatments of NRK‐52E cells. Following this, we assessed the generation of ROS using the ROS‐sensitive fluorescent dyes DCFH‐DA, and the fluorescence intensity was enhanced in the oxalate‐treated group (Figure 8C). Flow cytometry further confirmed that oxalate significantly induced apoptosis in HK‐2 and NRK‐52E cells (Figure 8D). Collectively, these findings highlight the cytotoxicity of oxalate in renal epithelial cells. Consistent with the results of GSE73680, the RNA‐Seq results demonstrated a marked reduction in the expression level of LAMA2 and CSNK1G3 in oxalate‐induced HK‐2 (Figure 8E,F) and NRK‐52E cells (Figure 8H,I). Likewise, the results of RT‐qPCR showed that LAMA2 and CSNK1G3 were downregulated in oxalate‐induced HK‐2 (Figure 8G) and NRK‐52E cells (Figure 8J).

## 4 Discussion

To the best of our knowledge, this is the first MR study to explore the causal relationships among food habits, plasma metabolites, and kidney stone formation. Additionally, eight food intake factors and 15 food liking factors were found to be significantly associated with the risk of kidney stones. The study also revealed mediation effects, in which certain plasma metabolites significantly mediated the relationship between dietary factors and the risk of kidney stones. Furthermore, this study identified 21 genes related to kidney stone formation through SMR analysis, shedding light on the genetic underpinnings of the disease. The downregulation of LAMA2 and CSNK1G3 in GSE73680 was consistent with the results of the SMR analysis. Lastly, we validated LAMA2 and CSNK1G3 expression with RNA‐Seq and in vitro assays. This comprehensive approach underscores the interplay among diet, metabolism, genetics, and kidney stone risk, highlighting potential prevention and treatment strategies.

Our analysis showed that the specific food intake, such as increased consumption of bread, was linked to a higher risk of kidney stones, whereas a preference for certain beverages like coffee and tea and fruits was associated with a lower risk of kidney stones. Notably, we found that genetic predisposition to coffee consumption, decaffeinated coffee consumption, and ground coffee consumption was associated with a decreased risk of calculus of kidney and ureter. Coffee consumption refers to the total intake of all coffee types, including regular, decaffeinated, instant, capsule, and ground coffee. Decaffeinated coffee refers to coffee with the most caffeine removed (< 3%), while ground coffee refers to coffee brewed from ground beans, excluding instant and capsule coffee. These are all associated with reduced kidney stone risk due to the beneficial effects of coffee's bioactive compounds, such as polyphenols and antioxidants, which may inhibit stone formation (Barghouthy et al. 2021a, 2021b; Peerapen et al. 2023). Caffeine can increase urine output and reduce CaOx supersaturation, while decaffeinated coffee retains other protective compounds (Costa‐Bauza et al. 2018; Ferraro et al. 2014). Additionally, ground coffee, being less processed, preserves more bioactive components, enhancing its protective effects (Zhang et al. 2024). Consistently, Yuan and others' findings suggest that increased coffee and caffeine intake was correlated with a lower risk of kidney stone formation using an MR study (OR = 0.60; 95% CI, 0.46–0.79; *p* < 0.001) (S. Yuan and Larsson 2022). Similarly, a previous study showed that the consumption of fresh fruit correlated inversely with kidney stone risk, which is consistent with our analysis (Turney et al. 2014).

Meanwhile, our mediation analysis revealed that certain metabolites play a role in kidney stones and mediate the relationship between diet and kidney stone risk. For example, we found that Histidine was associated with a decreased risk of kidney stones (OR = 0.93, 95% CI: 0.86–0.999, *p* = 0.049). Histidine is an essential amino acid, which means it cannot be synthesized by the body and must therefore be obtained from the diet (Brosnan and Brosnan 2020). Different amino acids, including Histidine, can influence urine chemistry, which in turn can affect the risk of kidney stone formation (Cao et al. 2023; Oliveira et al. 2019). Also, we found that metabolites like Mannose levels and Phosphate to mannose ratio were found to mediate the effects of food liking factors (e.g., bitter ale liking) on kidney stone risk. Phosphate, as a urinary constituent, can play a pivotal role in kidney stone formation, and elevated urinary phosphate can lead to increased saturation of calcium phosphate, potentially contributing to the formation of kidney stones (Alexander et al. 2022). Metabolites may mediate the diet–kidney stone risk relationship through altering urinary pH, inhibiting crystal growth, promoting diuresis, affecting oxalate metabolism, binding dietary calcium, modulating ion reabsorption, and exerting anti‐inflammatory effects (Fuster et al. 2022). Understanding how these and other metabolites mediate the dietary impact on kidney stone formation requires comprehensive metabolic profiling and analyses to pinpoint the exact metabolic pathways involved. Although the mediation proportions of these metabolites appear limited, they can be biologically meaningful (Li et al. 2024; Miao et al. 2025), as they may represent incremental contributions to disease risk within a broader network of interrelated factors given the complex and multifactorial nature of kidney stone formation, which involves multiple metabolic pathways, genetic factors, and environmental influences. For instance, Miao and others revealed that Mannose also mediated the protective effect of *s__Leuconostoc mesenteroides* (proportion mediated = 13.66%, *p* = 0.015) and *s__Turicibacter sp001543345* (proportion mediated = 13.03%, *p* = 0.015) against urinary stones, and Mannose was identified as a protective factor against stone formation in animal studies and clinical urine samples (Miao et al. 2025). Despite their modest size, these effects highlight critical biological processes that may influence kidney stone risk.

This study also highlighted the genetic underpinnings of the relationship between diet, metabolites, and kidney stones. Genes associated with the identified metabolites (e.g., CLDN10, OGFOD2, and GGT1) were found to have causal links to kidney stone formation. These genes could be involved in metabolic pathways that regulate the balance of minerals and other substances in the urine, affecting the likelihood of stone formation (Singh et al. 2022). Moreover, our analysis indicated that LAMA2 and CSNK1G3 are key genes that are downregulated in kidney stones. LAMA2, or laminin alpha 2, is a protein that is a part of the laminin family, and a proteomic‐based research strategy identified LAMA2 as a potential urinary‐specific biomarker for the medullary sponge kidney disease (Fabris et al. 2017). Consistently, previous studies have reported that the downregulation of genes like LAMA2 in CaOx stone patients suggests that alterations in extracellular matrix interactions and signaling pathways may contribute to kidney stone formation (Liang et al. 2020; Menezes et al. 2014). These mechanisms collectively point to a complex interplay between dietary habits, metabolic profiles, genetic predispositions, and biological pathways in influencing the kidney stone risk.

Furthermore, our pathway enrichment analysis demonstrated that LAMA2 and CSNK1G3 were linked to Mannose/Threonate and kidney stone formation through shared pathways, including the PI3K‐Akt signaling pathway, ECM–receptor interaction, and Focal adhesion. The PI3K‐Akt signaling pathway plays a critical role in the formation of kidney stones by regulating cellular processes, such as oxidative stress, apoptosis, autophagy, and inflammation (X. F. Wang et al. 2019; Xu et al. 2024; H. Yuan et al. 2021). For instance, Chen et al. found that miR‐155 facilitates CaOx crystal‐induced renal tubular epithelial cell injury via PI3K/Akt/−mediated autophagy (X. Chen et al. 2020). Recently, Xu and others suggested that umbelliferone attenuates CaOx crystal‐induced renal injury and inflammation by attenuating autophagy through the PI3K/Akt pathway (Xu et al. 2025). Focal adhesion is intimately linked to kidney stone formation as it governs the adhesion of CaOx crystals to renal tubular epithelial cells, a crucial initial step in stone formation (Li et al. 2025). It has been reported that miR‐223‐3p is significantly higher in urinary exosomes derived from CaOx stone patients and may affect stone formation via regulating focal adhesion and inflammation process (Yang et al. 2022).

In the evolving landscape of nutritional epidemiology, understanding the intricate web of causality between food habits and health outcomes remains a formidable challenge. The integration of diet, metabolomics, and genomics through MR in this study is more comprehensive than many traditional observational studies (Legay et al. 2023). Recent studies on food factors, including the consumption of specific items such as bread, coffee, tea, and fruits, have shed light on their potential roles as either risk or protective factors for kidney stones (Yitgin et al. 2023). Our MR results are credible due to the stringent assumptions testing, sensitivity analyses, replication across datasets, and biological plausibility; our approach may provide valuable insights into kidney stone interventions (Burgess et al. 2017). Through this mediation MR analysis, our study addresses a gap in the literature that often relies on associations without a clear causal direction between food metabolites and kidney stones. Our research also excels in the thorough examination of the biological mechanisms underlying kidney stone formation, delving into gene expression. We validated LAMA2 and CSNK1G3 expression with RNA‐Seq and in vitro assays in oxalate‐induced HK‐2 and NRK‐52E cells. This dual focus on the clinical evidence and biological underpinnings stands out as the cornerstone of our research.

It is important to acknowledge that, although our study has yielded significant findings, several limitations need to be carefully considered. (1) MR studies, while mitigating confounding factors, may still be influenced by unexplained factors. (2) The in vitro oxalate‐induced damage cell model does not directly mirror dietary oxalate intake in humans, and the identified target genes LAMA2 and CSNK1G3 require further research and verification through in vitro and in vivo experiments to ascertain feasibility and effectiveness. (3) Considering the role of tissue metabolites alongside plasma, sample selection bias may hinder comprehensiveness in understanding kidney stone formation, compounded by varying stone compositions. (4) The geographical limitation of the GWAS data, predominantly reflecting European dietary patterns, may constrain the generalizability of our findings to other populations, and caution should be exercised when extrapolating these results to other populations or contexts, such as those in Asia and Africa. (5) Mediation proportions for metabolites are modest in this study. The small effect sizes may reflect residual confounding or incomplete pathway coverage. Future studies incorporating broader metabolite profiling, proteomic markers, multi‐omics approaches, and improved statistical methods may provide deeper insights into the mediation effects and their clinical relevance.

## 5 Conclusion

Our multi‐omics investigation revealed how genetic predisposition, dietary habits, and plasma metabolites interact to influence kidney stone risk. Plasma metabolites (Mannose and Threonate) are key mediators in the diet–kidney stone nexus, and the downregulation of LAMA2 and CSNK1G3 is associated with a decreased risk of kidney stones, providing insights into potential therapeutic targets. However, reliance on publicly available datasets and limited population diversity are the limitations of this study. Future research should focus on validating the findings in diverse cohorts and exploring precision nutrition strategies for kidney stone prevention.

## Author Contributions

**Dawei Wang:** writing – original draft (lead). **Donghui Shi and Shuxin Si:** conducted the analysis. **Zijian Zhou and Fangxiu Luo:** designed the study and revised the paper.

## Ethics Statement

This study was approved by the Fudan Laboratory Animal Ethics Board.

## Consent

The authors have nothing to report.

## Conflicts of Interest

The authors declare no conflicts of interest.

## Supporting information


**Figure S1.** Pathway analysis of LAMA2/CSNK1G3 and targets of Mannose/Threonate. (A) The two‐dimensional molecular structures of Mannose/Threonate. (B) Venn diagram was acquired by taking the intersection of the targets of Mannose and Threonate in PharmMapper databases. (C) Pathway enrichment analyses were performed to elucidate the biological functions and signaling pathways associated with the targets of Mannose and Threonate. (D‐E) A protein–protein interaction (PPI) network for LAMA2/CSNK1G3 (D) and the pathway analyses (E) were constructed. (F) Pathway enrichment analyses of LAMA2 based on the Comparative Toxicogenomics Database (CTD).

**Table S1.** GWAS analysis of food intake using participants data from the UK Biobank.
**Table S2.** GWAS analysis of food liking using participants data from the UK Biobank.
**Table S3.** The list of 1400 blood metabolites.
**Table S4.** The primers used in this article.
**Table S5.** Heterogeneity analysis of food intake using participants data from the UK Biobank.
**Table S6.** Horizontal pleiotropy analysis of food intake with MR intercept values.
**Table S7.** Heterogeneity analysis of food liking using participants data from the UK Biobank.
**Table S8.** Horizontal pleiotropy analysis of food liking with MR intercept values.
**Table S9.** MR analysis of plasma metabolites.
**Table S10.** 50 plasma metabolites with a robust causal relationship with kidney stones.
**Table S11.** Seven pairs of associations between calculus of kidney and ureter‐associated food factors and metabolites.
**Table S12.** The multivariable MR analysis for correlated metabolites or dietary factors.
**Table S13.** The mediation of six food factor‐metabolites‐calculus combinations.
**Table S14.** The reverse MR analysis of food factors and kidney stones.
**Table S15.** Neighboring genes from IVs of 50 hub metabolites.
**Table S16.** SMR analysis using eQTL data from the eQTLgen Consortium identified 21 genes related to calculus of kidney and ureter.
**Table S17.** Differential gene expression analysis of the 21 genes from SMR analysis using the GSE73680 dataset.

## Data Availability

Data are provided within the manuscript or supplementary information files.
