# Supplementary material for: Dietary Influence on Urolithiasis Risk Mediated by Plasma Metabolites: A Mendelian Randomization and Experimental Study Linking Genes, Metabolites, and Clinical Outcomes
Source: Food Sci Nutr. 2025 Aug 19;13(8):e70800. doi: 10.1002/fsn3.70800 (PMC12364723; doi:10.1002/fsn3.70800)
Supplement: Supplementary file 1 — Figure S1. Pathway analysis of LAMA2/CSNK1G3 and targets of Mannose/Threonate. (A) The two‐dimensional molecular structures of Mannose/Threonate. (B) Venn diagram was acquired by taking the intersection of the targets of Mannose and Threonate in PharmMapper databases. (C) Pathway enrichment analyses were performed to elucidate the biological functions and signaling pathways associated with the targets of Mannose and Threonate. (D‐E) A protein–protein interaction (PPI) network for LAMA2/CSNK1G3 (D) and the pathway analyses (E) were constructed. (F) Pathway enrichment analyses of LAMA2 based on the Comparative Toxicogenomics Database (CTD). [file FSN3-13-e70800-s001.docx]

**Supplementary figures**

**
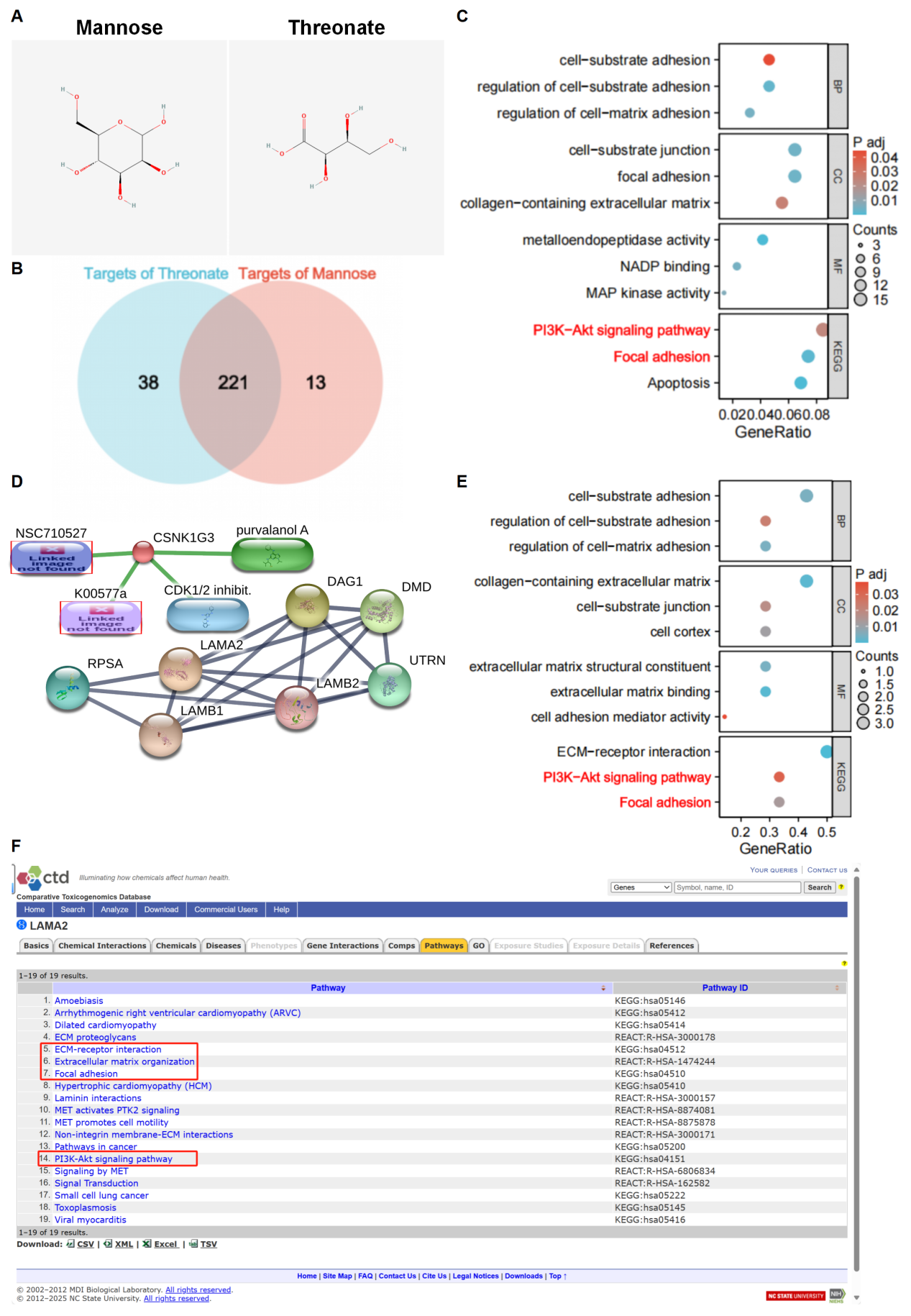
**

**Fig S1. Pathway analysis of LAMA2/CSNK1G3 and targets of Mannose/Threonate.** (A) The two-dimensional molecular structures of Mannose/Threonate. (B) Venn diagram was acquired by taking the intersection of the targets of Mannose and Threonate in PharmMapper databases. (C) Pathway enrichment analyses were performed to elucidate the biological functions and signaling pathways associated with the targets of Mannose and Threonate. (D-E) A protein-protein interaction (PPI) network for LAMA2/CSNK1G3 (D) and the pathway analyses (E) were constructed. (F) Pathway enrichment analyses of LAMA2 based on the Comparative Toxicogenomics Database (CTD).
